# Extracellular vesicles - on the cusp of a new language in the biological sciences

**DOI:** 10.20517/evcna.2023.18

**Published:** 2023-05-31

**Authors:** Graca Raposo, Philip D. Stahl

**Affiliations:** 1 Institut Curie, PSL Research University, CNRS, UMR144, Structure and Membrane Compartments, Paris 75005, France.; 2 Department of Cell Biology and Physiology, Washington University School of Medicine, St Louis, MO 63110, USA.

**Keywords:** Extracellular vesicles, biogenesis, intercellular communication, endocrine, homeostasis

## Abstract

Extracellular vesicles (EVs) play a key role both in physiological balance and homeostasis and in disease processes through their ability to participate in intercellular signaling and communication. An ever-expanding knowledge pool and a myriad of functional properties ascribed to EVs point to a new language of communication in biological systems that has opened a path for the discovery and implementation of novel diagnostic applications. EVs originate in the endosomal network and via non-random shedding from the plasma membrane by mechanisms that allow the packaging of functional cargoes, including proteins, lipids, and genetic materials. Deciphering the molecular mechanisms that govern packaging, secretion and targeted delivery of extracellular vesicle-borne cargo will be required to establish EVs as important signaling entities, especially when ascribing functional properties to a heterogeneous population of vesicles. Several molecular cascades operate within the endosomal network and at the plasma membrane that recognize and segregate cargos as a prelude to vesicle budding and release. EVs are transferred between cells and operate as vehicles in biological fluids within tissues and within the microenvironment where they are responsible for short- and long-range targeted information. In this review, we focus on the remarkable capacity of EVs to establish a dialogue between cells and within tissues, often operating in parallel to the endocrine system, we highlight selected examples of past and recent studies on the functions of EVs in health and disease.

## INTRODUCTION

A series of recent Nobel Prizes bookends the rapid progress in cell biology leading to our understanding of the biogenesis of extracellular vesicles and their promising but still uncharted role in human biology including medicine and plant science. Christian de Duve, who received the Prize for his discovery of the lysosome, speculated on the movement of materials into and out of cells in his depiction of a concept called exoplasmic space^[1]^. Exoplasmic space in eukaryotic cells is seen as an intracellular vacuolar space that is in immediate contact with extracellular space. At this interface, cells could both receive and discharge all manner of things. De Duve was aware of the role of cellular housekeeping in disease, and his early work led to the understanding of lysosomal storage diseases such as Pompe’s disease, in which cells cannot degrade glycogen and accumulate it within membrane-bound vesicles^[2]^. De Duve used the descriptor cellular defecation to describe a process where cells could rid themselves of unwanted material trapped within vesicular compartments, a problem of broad contemporary interest in studies of neurodegenerative and other diseases. The 1985 Nobel Prize to Joseph L. Goldstein and Michael S. Brown highlighted their work on cholesterol metabolism and focused on the mechanisms by which LDL particles entered cells^[3]^. A key finding was the discovery that signals are encoded in the cytoplasmic tails of receptors that engage a previously unknown sorting machinery that guides internalized receptors to their destination. Brown and Goldstein’s work and that of many others was a harbinger for the rise of a molecular understanding of membrane trafficking as we know it today. The molecular toolbox that opened a pathway to a broader understanding of membrane trafficking took advantage of the “awesome power of yeast genetics “and the work of Randy Schekman and his scientific offspring^[4]^. Schekman’s Noble Prize was awarded for his pioneering work on the genetic control of membrane trafficking that, like most Nobel Prizes, opened the gates of opportunity for generations of researchers, biological and biomedical. Now the challenge for the next generation is to build on these many accomplishments to develop or extend membrane trafficking models from intracellular to extracellular - how do cells use membrane trafficking pathways to allow cells and tissues to communicate with each other and how does this new understanding translate to diagnostics and therapeutics. What remains is a challenge for a new generation that will have a significant impact on our understanding of the human condition.

## DISCOVERED BY SERENDIPITY, EVS HAVE EVOLVED AS A PART OF A LARGER COMMUNICATION AND REGULATORY SYSTEM IN METAZOANS

Unlike most major discoveries in the biological sciences, the discovery of EVs as players in cell communication was revealed by serendipity and documented by careful follow-up research^[5]^. Now confirmed by many, the finding was revolutionary; vesicles released by one cell can deliver informational content to a second cell. Because the concept was new and not broadly tested and basically anathema to funding agencies, time was needed to broaden the concept. Over the past three decades, much has been accomplished and the concept is now widely accepted in scientific circles. The pathway from discovery to application is a long journey for most discoveries and longer for discoveries that break with commonly held views. Such is the case with EV-based signaling.

Cells from all three domains of life, Archaea, Bacteria and Eukarya, produce extracellular vesicles. In metazoans and multicellular plants, Extracellular Vesicle (EV)-based signaling allows cells to communicate with each other independently of cell-cell contact at short and long distances with different outcomes in tissue homeostasis and in disease^[6,7]^. Extracellular Vesicles released from the plasma membrane (microvesicles or ectosomes) and the endo-lysosomal system (exosomes) can be viewed as “miniature cells” devoid of nuclei and membrane-bound organelles but enriched in selected membrane proteins and lipids exposed at their membrane surfaces, and cytosolic components including both enzymatic and genetic material. EVs, once released from healthy and diseased cells, can reprogram recipient cells for “good or bad”^[8]^. EVs have been reported to be involved in virtually every aspect of human health and disease, and over the past years, diverse biological functions have been attributed to EVs depending on the cell types from which they were released. Among the first studies was the observation that EVs promote sperm cell motility by prostasomes (prostate cell-derived EVs)^[9]^. This early report did not assign an origin or a particular cargo associated with bioactive EVs. Studies by the teams of Rose Johnstone and Philip Stahl reported that reticulocytes release transferrin receptor-rich vesicles during the differentiation process^[10–13]^. The Harding *et al.* and the Pan and Johnstone^[11]^ papers demonstrated that the secreted vesicles were of endosomal origin and released upon fusion of multivesicular endosomes (MVEs) with the plasma membrane^[13]^. These studies opened the possibility of a new intracellular trafficking pathway that caught the interest of the cell biology community.

**Figure 1.**
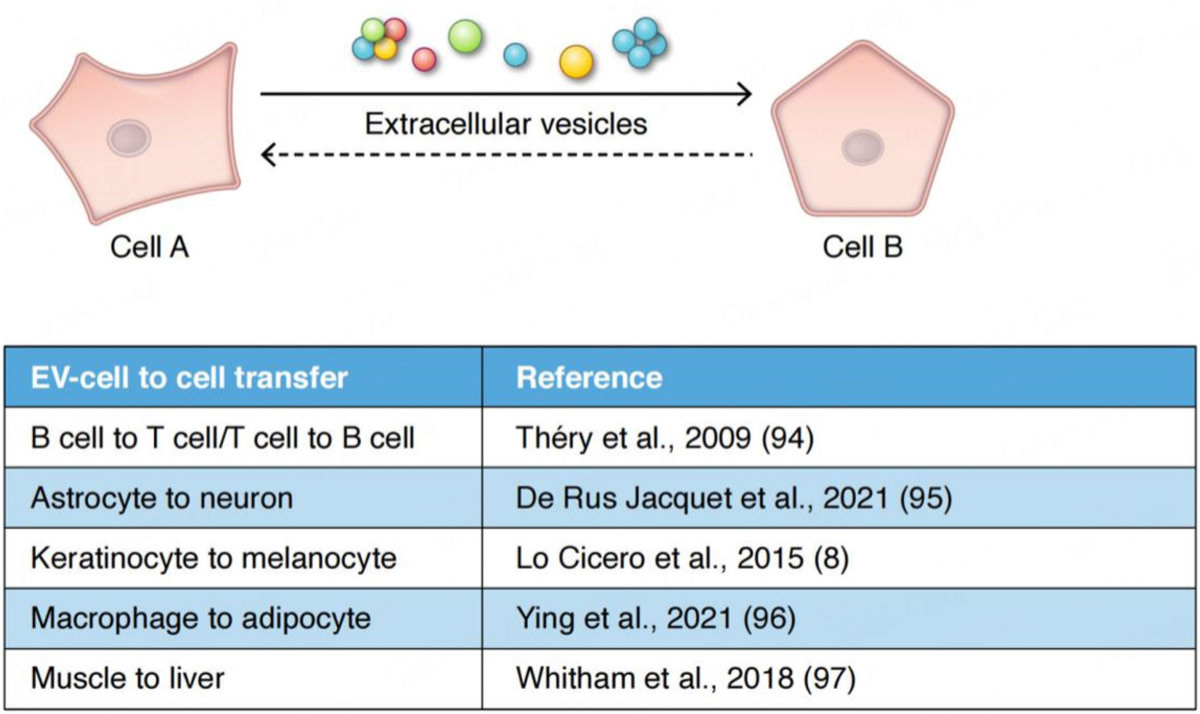
EV-based signaling. Information may be carried by individual EVs or via clusters of EVs. Clustered EVs may open the possibility of a quorum mechanism of signaling often found in bacterial communication. EV-based information exchange appears to influence all of the major organs and inter-organ homeostatic systems in metazoans, including the immune system^[94]^, the nervous system^[95]^, the skin^[8]^, and the endocrine system^[96]^. Overall metabolism may be influenced by EVs released from muscle upon exercise^[97]^. In the endocrine system, the interplay between the macrophage and the adipocyte via EVs in the regulation of insulin responsivity represents one example of a close collaboration between endocrine function and EVs^[96]^. In these various examples, is the conversation or information transfer between cells or tissues (as depicted above) a one-way signaling mechanism or is the information transfer two-way? If information transfer were a two-way mechanism, what would be the nature of the return pathway-vesicular or humoral? What are the complex feedback control mechanisms in play in participating cells? It remains to be seen.

In metazoans, EVs impact communication and exchange among cells within tissues, within the circulation, and even at the interface with the external environment such as in the microbiome^[7,14]^. A variety of reports now confirm that EVs are players in the information exchange that occurs between cells at the tissue level, in the nervous system (e.g., via exchange among and between astrocytes, glia and neurons)^[15]^, in the immune system (as initially revealed by the early pioneering work on exosomes and antigen presentation^[16,17]^) between B cell, T cells and dendritic cells, and in the integumentary system between keratinocytes, melanocytes and fibroblasts among others^[18,19]^. Recent studies suggest that EVs may play an important, if unforeseen, role in homeostasis in the endocrine system. EVs may deliver informational content between tissues and cells that influence their subsequent responsivity to insulin^[20,21]^. Exercise studies in particular have drawn much attention where EVs released in response to exercise appear to have a broad influence on metabolism^[22]^. Muscle releases exerkines that may be packaged in EVs^[23]^. Similar to paracrine signaling, but at longer distances, EVs may operate in parallel with the endocrine system, where EVs carry integrative messages connecting tissues with tissues (e.g., adipose EVs and the brain) [Figure 1]^[21]^. The endocrine system evolved in metazoans, in part, as a spin-off from the nervous system, whereas EV signaling probably evolved much earlier. Endocrine signaling and EV signaling, therefore, evolved in parallel. EVs may play a role as an “invisible hand” that links the endocrine system to what is happening at the tissue level.

Early reports revealed that cells release EVs, exosomes, in particular, for disposal of “unwanted” cellular components^[24]^ as part of cellular housekeeping or as content that can be used for trophic support^[25]^. EVs can operate as “independent metabolic units” where they can influence the milieu of the microenvironment or be exploited as signaling units between cells^[26]^. At the tissue and organism levels, EVs can induce cellular responses as initially reported in the immune system^[17]^ and in the central nervous system^[27]^ or in the skin^[18]^, among others. In the immune system, the ability of B lymphocytes to stimulate T cell proliferation^[16]^ and the findings that EVs secreted by dendritic cells injected into mice bearing tumors can suppress not only tumor growth but also tumor eradication^[17]^ has been exploited to enhance anti-tumor immune responses in patients and influenced immunotherapy protocols^[28,29]^.

A growing interest has focused on regenerative properties associated with EVs released from mesenchymal stem cells^[30]^. Various reports have shown promising results in wound healing and in recovery from ischemia and tissue fibrosis, among others^[31,32]^. Other interesting findings are the functions of placental-derived EVs during pregnancy, from placenta establishment to maternal immune tolerance towards the fetus and protection against viral infections^[33]^. In pathological scenarios, on the “bad side of the coin” as it were, EVs can induce tolerance in the immune system and promote tumor progression by being involved in organotropism and metastasis^[34]^ or sustain infectious or inflammatory states^[35]^. EVs may be involved in pathogen transmission in neurodegenerative disease by ferrying prions and other protein cargo involved in these diseases^[36]^. Another example is that in viral infections such as SARS Cov2, EVs have been shown to express the spike protein on their surfaces, thereby blocking the effectiveness of endogenous neutralizing antibodies^[37]^. Despite all the aforementioned downsides, whether delineating EV function in normal tissue or in diseased states, expanding our knowledge pool carries an enormous payoff as it opens avenues for varied diagnostic and therapeutic applications^[38]^. Several bench-to-the-bedside applications of EVs are now envisioned and exploited by established and emerging startups (https://bioinformant.com/top-exosome-companies/)

## REGULATED EV ASSEMBLY AND RELEASE APPEARS TO BE A UNIVERSAL CHARACTERISTIC AMONG EUCARYOTIC CELLS

The goal of EV research is to achieve a comprehensive understanding of the depth and breadth of “EV Biology” in all forms of life. Several large hurdles remain to capture this goal. These include vesicle heterogeneity and its origins, isolation, using novel biophysical and biochemical methods and compositional characterization (e.g., is there a single EV species with all the features necessary to mediate cell-to-cell signaling)^[7,39]^. In connection with the latter is signaling or messaging due to a quorum effect as found in bacteria. Lastly, assigning functions to specific EVs or groups of EVs is a universal goal among EV research enthusiasts while developing an understanding of their biogenesis and targeting. EV nomenclature has been and is a continuing challenge. The commonly used EV nomenclature encompasses both endolysosome derived- and plasma membrane derived-vesicles^[39]^. Complexity is further increased by the findings that EVs released from cells are neither dispatched indiscriminately from endosomes nor random sites in the plasma membrane, but rather from specific membrane domains including cell protrusions and primary cilia^[40,41]^. Additional types of subcellular-derived structures are migrasomes^[42]^, apoptotic bodies^[43]^, and midbody remnants^[44]^ that can also be considered EVs. A new particle, the Exomere, possibly not of endosome or plasma membrane origin, has been recently described and reported to transfer cargo^[45]^, although no biogenetic pathway(s) has been assigned to it [Table 1]. Lastly, the molecules surrounding EVs, so-called the corona^[46]^, may influence EV function. They are not merely contaminants as EVs from blood plasma surrounded by such a “corona” were functional by inducing an increased expression of TNF-α, IL-6, CD83, CD86 and HLA-DR in human monocyte-derived dendritic cells^[46]^; other proteins associated with the outer leaflet of EVs, such as tetherin, allow EVs to cluster to potentially increase their affinity for a target^[47]^. In short, massive heterogeneity requires the development of optimized isolation and quantification procedures to standardize reporting of results and to better assign the observed functions to different types of vesicles and vesicle subpopulations^[39]^. Currently, several methods are being used depending on the starting samples (e.g., conditioned media from cell cultures, biological fluids, tissues, model organisms), the paucity of material that can often be limiting depending on the applications. When handling conditioned media from cultured cells, differential ultracentrifugation is generally used as a first approach followed by a thorough characterization using western blotting, nanoparticle tracking, and importantly, electron microscopy, the only method with enough resolution to visualize small membrane-bound vesicles and even their origin (namely, budding from the plasma membrane or endosome fusion with the plasma membrane)^[48,16,49]^. Floatation gradients (sucrose, Percoll) are also generally performed to show the vesicular nature of the isolates, including their density^[48]^. Moreover, possible heterogeneity can be appreciated by monitoring the distribution of marker proteins using western blot. Interestingly, mass spectrometry reveals several protein components that can be common to exosomes and ectosomes from different cell types in addition to cell type-specific proteins^[50]^. These methods are certainly lengthy and difficult to adapt to small amounts of material. Other methods include Size Exclusion Chromatography (SEC)^[51]^ and Asymmetrical-flow field-flow fractionation^[52]^ that optimally maintain EV function. Immunoaffinity-based methods, ultrafiltration, anion exchange chromatography, and microfluidics have promising possibilities^[53]^. All these methods have advantages and disadvantages and allow the isolation of EVs with different features such as density, size, and charge. The characterization of recovered EV fractions and description of the methods used is essential in the reporting of results (see MISEV Guidelines)^[39]^.

## ELUCIDATING THE MOLECULAR MECHANISMS THAT OVERSEE EV BIOGENESIS AND SECRETION-A MAJOR CHALLENGE FOR THE BIOMEDICAL RESEARCH COMMUNITY

**Figure 2.**
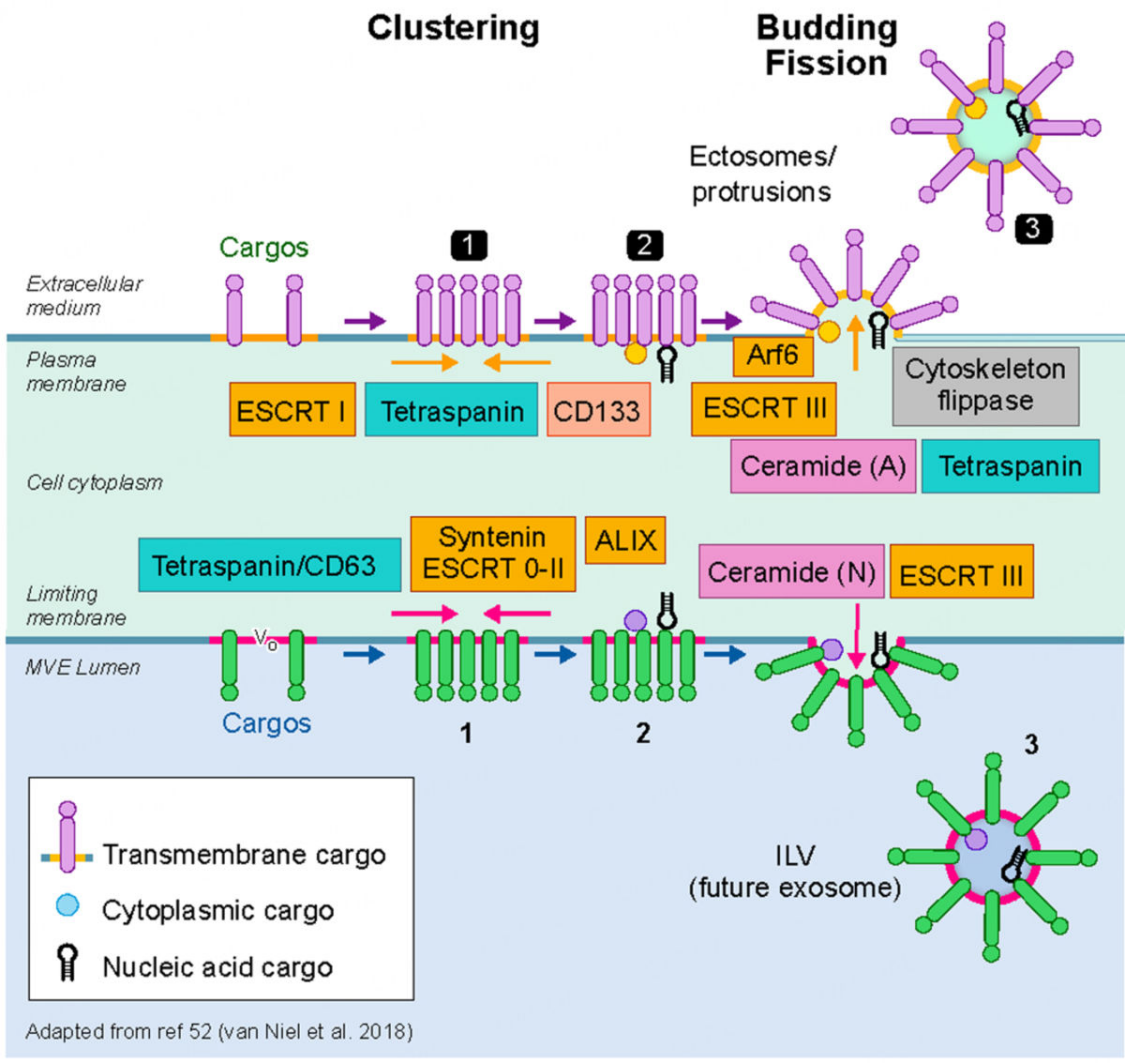
Biogenesis of extracellular vesicles. A current view of exosome and ectosome biogenetic mechanisms is shown at the bottom and top half of the image, respectively. The formation and assembly of exosomes begins in a specialized multivesicular endosome (MVE) where intraluminal vesicles (ILV), enriched in cargo carriers (tetraspanins such as CD63) and deliverables, among other generic markers, are formed by the inward vesiculation of a cholesterol, sphingomyelin enriched microdomain. The process is driven by members of the ESCRT family of proteins (ESCRT 0-II; ESCRT3) and/or by the enrichment of ceramide and other membrane bending factors via the action of sphingomyelinase. The V0 subunit of the V-ATPase may also play a role^[98]^. Ectosomes (top half of the image) are formed at the plasma membrane by a variety of mechanisms including the ESCRTs often interacting with ARRDC1^[56]^and CD133^[61]^, the latter shown to be required for small ectosome biogenesis from microvilli in drosophila epithelium. Arf6 plays a role in some forms of ectosome secretion^[60]^. Recent work also shows that I-Bar domain proteins may play a role in the membrane curvature required for ectosome biogenesis and release^[99]^.

The intracellular mechanisms that govern the biogenesis and secretion of EVs are clearly complex and overlapping. Moreover, our understanding of their depth and scope is clearly incomplete, even at this stage in the work. An important goal in unraveling the molecular mechanisms of EV biogenesis, secretion and targeting is to eventually understand EV function and whether one can selectively interfere with EV generation, release, and targeting. Over 20 years ago, the discovery of the ESCRT machinery in yeast and in mammalian cells revealed a first hint that led ultimately to a general understanding of how cargo is sorted within endosomes and incorporated into newly formed intraluminal vesicles that could either be released as exosomes or transferred to lysosomes for degradation^[54,55]^. Although these initial observations offered great progress, the overall picture of EV biogenesis remains a sketch and appears much more complex than initially conceived^[6]^. The biogenesis of extracellular vesicle subpopulations relies on several steps that are common to exosomes and ectosomes, the latter referring to vesicles emanating from the plasma membrane. A membrane microdomain is formed engaging specific sets of cargoes, including a portion of cytosol, after which the domain buds and forms a neck that fissions to release a vesicle either directly in the extracellular milieu, if happening at the plasma membrane, or into the lumen of a multivesicular body. If ESCRT subunits can modulate the sorting of certain cargoes leading to the formation of a subpopulation of intraluminal vesicles within endosomes, then it seems reasonable and perhaps likely that selected ESCRT subunits may also operate at the plasma membrane^[56]^. There are other protein components and lipid-based mechanisms that operate at both sites [Figure 2]. Ceramide production has long been shown to be essential for the sorting of proteolipids in oligodendrocytes^[57]^. Tetraspanins, including CD63 that form microdomains at the endosomal membrane, are required for the sorting of particular cargoes^[58]^. Moreover, syntenin and syndecans, together with Alix, an ESCRT accessory protein, are also important for ILV formation and therefore for exosome secretion^[59]^. Despite that some components of the sorting machinery appear to act preferentially at the plasma membrane rather than on the endosomal system (ARF6, CD133/prominin)^[60,61]^, the redundancy built into these various molecular machineries together creates a roadblock to experimentally modulate their biogenesis, release and therefore their function. One should consider the cargo of interest that recruits and pairs with particular machinery for sorting at the endosomes or at the plasma membrane.

Because of the built-in redundancy, interfering with any single cog in the machinery is insufficient to modulate EV secretion. While sorting machineries are thought to cluster and enrich cargoes on microdomains that bud into EVs, cargoes, and their post-translational modifications, recruit the appropriate sorting machineries. EVs, once released from cells, are targeted to their physiologically relevant recipient cells to elicit cellular responses. EVs can also be internalized by reticuloendothelial cells such as macrophages. How EVs find their targets and how their contents are specifically delivered and processed is far from being elucidated. Depending on the cell type and cargo sequestered, the mechanisms may vary. The diversity of mechanisms correlates with the heterogeneity in size and composition of ILVs that give rise to different exosome subpopulations that may execute different functions^[7]^.

Cargo plays a role. Expression of a single cargo protein targeted to MVEs can turn the recipient endosomes into secretory endosomes. As an example, the expression of MHC class II in Hela cells allows the recruitment of the small GTPase Rab27 to MVBs while increasing the release of MHC II-positive exosomes^[62]^. Interestingly, Rab27a is associated with most, if not all, secretory lysosomes (so-called Lysosome Related Organelles), which indicates that secretory MVEs display features shared with organelles of the LRO family such as melanosomes, cytolytic and basophilic granules, mast cell granules, platelet dense granules^[63]^. This suggests that secretory MVEs correspond to a subpopulation of *bona fide* MVEs whose normal or main fate is to fuse with lysosomes leading to the degradation of endogenous or internalized components. Subpopulations of MVEs have been reported in different cell types. Of interest, cholesterol-rich MVEs appear to be more prone to exocytosis^[64]^. Subpopulations of secretory MVEs can also be generated upon cell-cell interactions, as shown for immune cells^[64,65]^. Recent studies strongly support the notion that secretory MVEs acquire the machineries required for transport/fusion with the plasma membrane through tight contacts with other organelles and, in particular, the endoplasmic reticulum that allows the small GTPase Rab7 to enter a cascade leading to the activation of the above mentioned Rab27^[66]^. Therefore, MVEs correspond to a subpopulation of endosomes that carry different effectors to traffic close to the cell surface. The release of exosomes versus plasma membrane-derived ectosomes is subject to additional regulatory steps, such as the targeting of the endosomal compartments to the plasma membrane and their fusion. Another way to further decipher the mechanisms leading to MVB secretion is to exploit specific reporters such as CD63-pH-fluorin to visualize and define the composition of secretory MVBs that can be used both “in vitro” and “*in vivo*” in model organisms^[67,25]^.

## CARGOES AND SORTING MACHINERIES- DOES CARGO BEGET EV BIOGENESIS?

As alluded to above, expression of specific cargoes (e.g., MHC II molecules) can lead to a robust increase in the production of EVs. While sorting machineries are thought to cluster and enrich cargoes on microdomains that bud into EVs, cargoes and their post-translational modification recruits these sorting machineries. Understanding the selective packaging of membrane and cytosolic proteins and genetic material into ILVs to be secreted as exosomes or to discrete domains of the plasma membrane to be released as ectosomes opens the way to understanding how composition can be modulated^[7]^. Basically, elucidating the mechanistic basis of EV biogenesis opens the door to understanding EV secretion and the targeting of signaling molecules to recipient cells. Cargoes that are selected by the aforementioned molecular machineries can be post-translationally modified for selective recognition^[68]^. Ubiquitination does not appear to be required for sorting of MHC class II molecules to secretory MVEs, although ubiquitinated proteins are commonly found in EVs^[69]^. Interestingly, ESCRT components that sort proteins in MVEs contain Ubiquitin Recognizing Motifs^[54]^. Not ruling out ubiquitin completely, there are many forms of ubiquitin conjugation including mono-ubiquitination involved in targeting CD133 to EVs^[70]^. Other post-translational modifications, such as palmitoylation and farnesylation, which bring proteins to lipid rafts, are also implicated in EV biogenesis^[68]^. The selective packaging of RNA into nascent EVs has seen significant progress with the discovery that some miRNAs contain a targeting sequence recognized by a sumoylated hnRNPA2B1^[71]^ and that YBX-1proteins^[72]^ may selectively recruit specific miRNAs into newly forming exosomes. Given the wide collection of RNA molecules that may be packaged as cargoes, different sorting machineries may be exploited possibly by post-translational modification (e.g., ubiquitination, palmitoylation, *etc*.)^[73]^. Based on the potentially wide variety of sorting mechanisms and their role in biogenesis, several questions arise: do all sorting mechanisms act on the same compartments, do they act sequentially at different stages in maturation of these compartments, or do they act on distinct compartments with unique features and fates. Of note, in endosomes, the cargoes and the sorting machineries could also recruit or exclude additional machineries that regulate lysosomal fusion, MVE transport to the plasma membrane, or fusion^[7]^. One should also keep in mind that the budding step by itself will change the composition of the limiting membrane and could lead to the recruitment of these regulatory components. Lastly, EVs are secreted by polarized epithelia from both the apical side as well as the basolateral domain^[74]^. Recent work suggests that different EV sorting and secretion mechanisms may be at play at these two locations^[75]^. Apical secretion and basolateral secretion of EVs are likely to have widely disparate functions.

## CELL-CELL CONTACTS AND EVS WITHIN TISSUES, IMPACT OF THE MICROENVIRONMENT

Close encounters within the tight quarters of the tissue environment raise many questions about how cells communicate with each other: humoral (paracrine, autocrine), tubule (nanotubes), or vesicular^[76,77]^. While the budding from the plasma membrane seems to be a relatively simple event and exosome secretion a more regulated process, tracking the role of EVs in a 3D microenvironment raises key questions, including cell type heterogeneity and the effect of external factors^[7,78]^. Within tissues, cells in contact with each other can communicate via receptors and by direct contacts that can be maintained by filopodia and nanotubes^[77]^. In light of this, the need for a cell-free membrane-bound vesicle may be questionable. However, the very selective packaging of cargo within EVs, independently of their origin, is likely to allow for communication at short distances between cells while allowing for longer-range signaling within a tissue^[61]^. Additionally, physical parameters such as external pH, osmotic pressure, and physical constraints due to the matrix structure of 3D environment including mechanical stress may affect EV secretion and targeting^[79]^.

Several examples may serve as a kind of intellectual *hors d’oeuvre* to capture exciting developments in EVs operating in the microenvironment. Contacts between cells, such as that observed during immune synapse formation, elicit an increase in EV secretion and remodeling of the endocytic organelle to generate a subpopulation of secretory MVEs^[65]^. An interesting recent report highlights the potential importance of EVs in the immunological synapse^[80]^. Lanna *et al.* showed that antigen-presenting cells (APCs) extend the lifespan of T cells that they form synapses with by transferring telomeric DNA from the APC to the T cell via EVs. Telomeric DNA in APCs is trimmed away from its chromosomal localization and packaged in EVs that are then secreted and delivered to the recipient T cells. These findings may be a harbinger of a new understanding of the role of EV information transfer during close encounters, in this case in the immunological synapse, but potentially in other physiologically important cell interactions such as the retina^[81]^.

EVs can also directly interact with and remodel the cell environment. As an example, melanoma-derived EVs can interact physically with collagen. EVs modulate the extracellular matrix which could have consequences on their diffusion in the tumor microenvironment and therefore affect the capacity of EVs to interact with the different cell types that constitute the tumor microenvironment^[82]^.

## SYSTEMS TO SIMULATE EV SIGNALING “IN VITRO” AND “IN VIVO” - DELINEATING THEIR ROLE IN TISSUE PHYSIOLOGY AND PATHOPHYSIOLOGY

**Figure 3.**
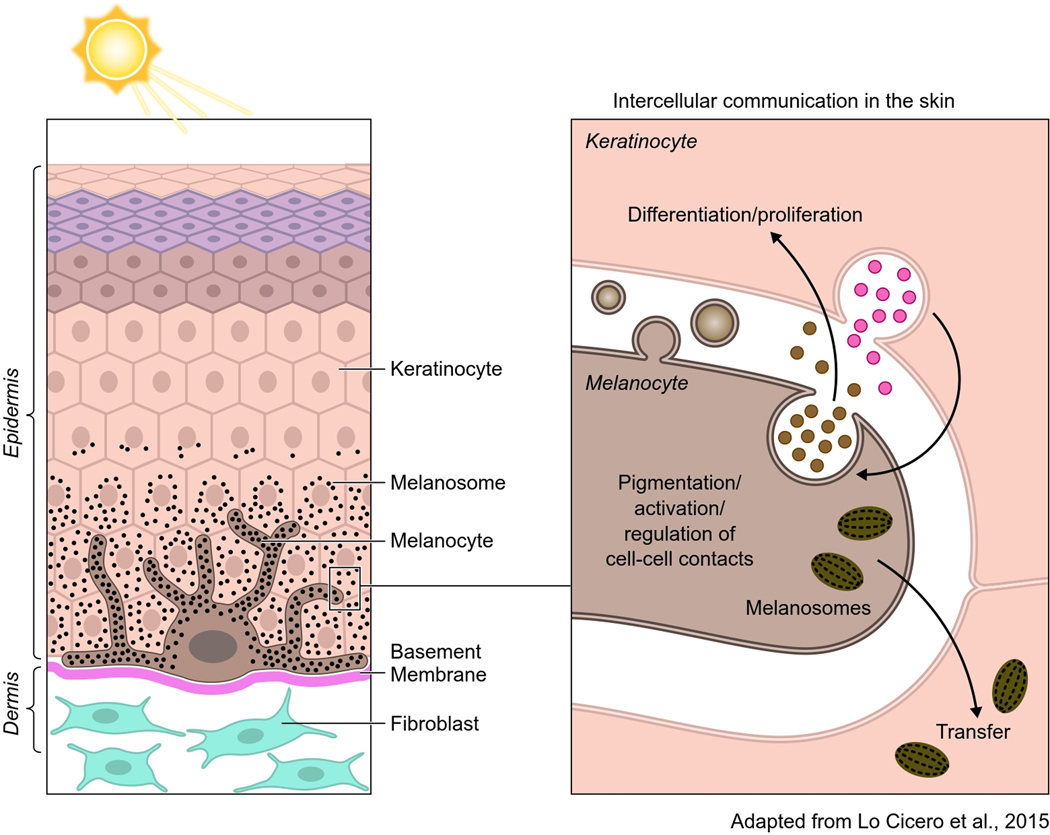
In the skin epidermis, melanocytes produce the pigment melanin in organelles called melanosomes (black dots). Melanocytes extend their dendrites contact with basal keratinocytes and transfer the pigment that accumulates in the keratinocyte to color the skin and to photoprotect the skin against ionizing radiations (UVB). Although melanocytes and keratinocytes communicate by direct contact, they also communicate via EVs. Exosomes, secreted by keratinocytes, modulate pigmentation and certainly melanocyte function. Melanocytes also secrete EVs from the endosomal system and plasma membrane that are likely to be involved in keratinocyte behavior (e.g., differentiation, proliferation, and melanosome transfer).

One of the goals of EV research is to understand their role in tissue homeostasis. To achieve this goal, investigators have developed “in vitro” approaches simulating organ environments, including the heart, adipose tissue, and skin, among others. More recently, iPSC-derived organoids have been developed to simulate various tissue environments, including the brain which have been particularly illuminating^[83]^. Skin, the largest organ in the organism, has appeared as a fascinating puzzle to understand EV biology and their functions in homeostasis and disease^[84]^. In the skin epidermis, melanocytes embrace around forty keratinocytes with their extended dendrites [Figure 3]. They are in close contact with each other establishing a so-called “pigmentary synapse” in which caveolae play an essential role in mechano-signaling and pigment transfer^[85]^. Keratinocytes and melanocytes secrete factors that are required to control skin cell function^[86]^. In addition to soluble factors, they also secrete EVs carrying proteins, lipids and genetic material that can be involved in controlling cell-cell contacts and several aspects of skin homeostasis [Figure 3]. Keratinocytes secrete EVs with features of exosomes, enclosing specific miRNAs that are targeted to the melanocytes to control the expression of melanosomal proteins and, consequently, modulate pigmentation^[18]^. In a feedback loop, melanocytes secrete a heterogeneous population of EVs that are likely to regulate keratinocyte biology and functions (our unpublished studies). Any deregulation in these pathways may underlay pigment disorders^[87]^. Moreover, they may be involved in pigmentary disorders in which intercellular communication is altered and in skin melanoma and carcinoma where they potentially contribute to the progression of metastasis^[88,89]^. Other cells present in the skin, such as dermal fibroblasts, can also secrete EVs or be the recipient of keratinocyte and melanocyte EVs, establishing a communication network within a complex tissue^[90]^. Clearly, these are important components of the overall homeostasis of the skin. EVs and their signaling capacities could be used in therapeutic strategies in skin regenerative medicine by exploiting stem cells^[91]^.

## CONCLUSIONS

Membrane trafficking came of age, starting with the description of the secretory pathway and the lysosome in the middle part of the last century, as set out in the Introduction. The various Nobel prizes awarded along the way serve as markers for the amazing progress achieved during this period and up to time present. With the discovery that cells can selectively package and secrete content in vesicular form and that the secreted vesicles can act in cell signaling, a new chapter in membrane trafficking has emerged. Vesicle secretion by bacteria^[92]^ and eucaryotic cells has been known for years, but the idea that signaling information can be transferred between and among cells has introduced a new lens in viewing physiology in animals and plants^[93]^. Consequently, the entire cell biology community, along with researchers in physiology and pathophysiology, have been sparked by curiosity - how are information-containing vesicles formed and how is information packaged; moreover, what are the downstream targets of such vesicles and what is the new molecular language of recognition that allows delivery of information to target cells. The puzzle has many components-at this point, we know that information containing vesicles can be generated at the plasma membrane or are born in the endocytosis-lysosome network in the form of multivesicular endosomes (MVE) or bodies that may form *de novo*, as compared to MVEs that target their content to the lysosome for degradation^[6,7]^. Unlike the adage, “all roads lead to Rome”, there are likely many pathways for packaging informational content either via the MVE mechanism or via budding from the plasma membrane. It is clear that a number of Rab GTPases, long known to regulate membrane trafficking in cells, are involved, as well as cascades where early-acting Rab/GTPases are connected with the activation of downstream Rabs, ultimately leading to the activation of Rab27 which now seems established as a common Rab regulator of fusion with the plasma membrane^[66]^.

EV biology. What are the major challenges looking ahead and will we be prepared for what appears over the horizon? First, we do not have a complete picture of the mechanisms of cargo packaging and the loading of cargo into vesicles embedded with cognate directional information that will guide their fate. More needs to be done on the mechanisms by which vesicles interact with target cells that allow informational content to enter the target cell in a biologically active form and at what site the information is translated. Lastly, vesicles are heterogeneous. Why? Does heterogeneity reflect different biogenetic pathways and separate content or is this due to biological variation? More needs to be done to separate and analyze populations of EVs. New technology will carry the day. Will EVs play a central role in integrating development and physiology? These are central questions that will open opportunities for a new generation of biomedical scientists. What are the expectations that EV biology will break barriers in the future? The epigenome, once inaccessible to manipulation, especially in ova, may now be accessible by EV technology. Will the Weismann Barrier be breached? How does virus spread in the body and are EVs being hijacked by virus as some reports suggest, to facilitate spread? Is Long COVID due, in part, to the distribution of virus across biological barriers? Lastly, will EV applications flourish? EV technology may change the practice of medicine through diagnostics and therapeutics. EV applications in agriculture are still in a nascent stage, and undoubtedly, more will come. The long and short of this narrative is that starting from a key basic science discovery, a new era of cell communication has emerged that will reveal more of what is novel about the human condition.

## FUTURE PERSPECTIVES AND ROADMAP FOR THE NEXT GENERATION

### Finding the evolutionary origins of EV biogenesis and secretion

The mechanisms of vesicle formation and secretion in prokaryotes are undoubtedly the forerunners of EV secretion in eukaryotes. Comparative analyses of EVs from the three kingdoms will be revealing.

### Elucidating the biogenesis and secretion of EVs in eukaryotic cells

Understanding the biogenesis of multivesicular bodies harboring exosomes and the assembly of nascent EVs at the plasma membrane will be essential to the goal of transferring EV technology to diagnostics and therapeutics. There are likely numerous junctions and inputs along the various trafficking pathways, including different modes of packaging: at the cell surface; within endosomes; in autophagosomes; and even in nuclear outer membranes. How would the assembly process be regulated at the transcriptional level? Do EV assembly and discharge pathways operate as escalators that constantly move along the secretory pathway with or without cargo or would the assembly process be tied to the production of cargo or cargo carriers?

### Cognition and reception

Specificity is key to EV-dependent communication. Signal transmission by EVs will require some form of molecular recognition of EVs by target cells. How recognition molecules are packaged into EVs that also contain informational content will remain a central but challenging question. Recognition of EVs by target cells might be achieved by low affinity interactions enhanced by vesicle clustering akin to a quorum effect found in bacteria. Understanding the modes of access to target cells will be essential to the development of EV-based therapeutics.

### Methods, models and the microenvironment

Tracking EV discharge and delivery at the single-cell level in the microenvironment will reveal the role of EVs in the creation of so-called niche or pre-metastatic environments. Methods to study EVs at the single-vesicle level, including improved imaging technologies and innovative *in vitro* reconstitution techniques, will be required to examine all aspects of EV biogenesis. Exploitation of organoid technologies will be needed to create novel microenvironmental simulations. Lastly, animal models will be needed to monitor EV traffic and exchange in real time, opening a new level of understanding of the interplay between EV signaling and the endocrine system.

### Diagnostic and therapeutic applications

A major challenge in the coming decades is the application of EV-based technology to overall human health. Advances in EV-based diagnostics will depend on the discovery of unique EV markers that would serve as molecular signatures that report on both disease and physiological states. Finally, reconstitution or packaging of targeting molecules and informational content into deliverable EVs will open up a new era in therapeutics.

## Figures and Tables

**Table 1.** Examples of subpopulations of Extracellular Vesicles

Extracellular Vesicles	Size	Markers	Biogenesis

Exosomes	30 nm-110 nm	Tetraspanins (CD63) ESCRT complex subunits and associated proteins (Tsg101, Alix) Syntenin	Correspond to the intraluminal vesicles of MVEs. They are generated by inward budding of the endosomal membrane and they are secreted upon fusion of MVEs with the plasma membrane
Ectosomes (microvesicles, oncosomes)	50 nm-10,000 nm	Annexin A1, ARF6	Generated by outward budding from the plasma membane. In some cell systems, they can be formed at specific sites such as membrane protrusions
Migrasomes	500 nm-3,000 nm	TSPAN4	Generated during cell migration from long retraction fibers. “pomegranate-like structures”, morphologically similar to MVEs, are formed on these fibers and then released
Secretory autophagosomes/Amphisomes	Not determined	LC3	Generated through macroautophagy (secretory autophagosomes) or fusion of autophagosomes and MVEs
Exomeres	< 50 nm	Enriched in proteins Involved in metabolic pathways	Unknown but defined as “non-membranous”
Apoptotic bodies	50 nm-5,000 nm	Phosphatidylserine	Released from apoptotic cells upon activation of apoptosis-related transduction pathways
Released Midbodies	200 nm-600 nm	Tubulin MKLP2 CEP55	Released by dividing cells during cytokinesis and can induce cell proliferation once uptaken by recipient cells

## Data Availability

Not applicable.
